# Analysis of orthopaedic private healthcare patterns in England: A potential emerging two-tier system

**DOI:** 10.1016/j.puhip.2024.100578

**Published:** 2024-12-24

**Authors:** Lucy McCann, Ian Holdroyd, Rowan Emberson, Helena Painter, John Ford

**Affiliations:** a Wolfson Institute of Population Health, Queen Mary University of London, London, UK; b Department of Public Health and Primary Care, University of Cambridge, Cambridge, UK; c Barts and the London School of Medicine and Dentistry, Queen Mary University, London, UK

**Keywords:** Private healthcare, National health service, Waiting times, Health inequity, Geographic disparities

## Abstract

**Objectives:**

Private healthcare is a rapidly growing industry in the UK, particularly for surgical procedures, due to extensive waiting times in publicly funded health care. The NHS also commissions private healthcare to provide procedures for NHS patients to alleviate waiting times. We aimed to explore the trends and geographical variations between the North and South of England in privately funded and NHS-funded privately delivered orthopaedic procedures compared to NHS waiting times.

**Study design:**

A longitudinal study using quarterly national data between 2019 and 2023 in England.

**Methods:**

We analysed orthopaedic surgical volumes per 10,000 people using Private Healthcare Information Network data in England from 2019 to 2023 and compared them with waiting times in publicly funded health care provided by the NHS. We stratified by geographical location and time period to compare the North-South divide in England.

**Results:**

The south of England performed almost double the number of privately funded procedures (23 vs 12/10,000), but there were fewer NHS-funded private procedures (40 vs 45/10,000). The north of England has consistently shorter waiting times than the South, with considerable variation across regions. London had fewer NHS-funded procedures compared to other regions.

**Conclusions:**

The time-trend patterns indicate considerable geographical inequalities of access to orthopaedic private healthcare between regions within England, with a potential emergence of a two-tier healthcare system. Relying on the private sector to reduce waiting lists, without oversight, may exacerbate regional and socioeconomic differences. Policymakers should consider how the unequal distribution of funding and NHS-funded procedures could perpetuate inequalities.

## 1 Introduction

Private healthcare is a rapidly growing industry in the UK [1]. In 2022, the three largest UK private health insurance providers registered half a million new customers [2], and a report by the Institute of Public Policy Research (IPPR) showed that the UK has the fastest-growing money spent on private insurance and out-of-pocket healthcare of all G7 nations [1]. People are increasingly turning towards the private sector for surgical procedures as waiting lists have become longer than ever before, exacerbated by the Covid-19 pandemic. Across the pandemic period, waiting lists for elective surgical and consultant-led care increased from 4.4 to 7.5 million patients, with no signs of improvement [3].

There are three main types of funding for healthcare procedures in England: NHS-funded in a public facility, NHS-commissioned in a private facility (NHS-funded privately delivered procedures), and privately funded privately delivered procedures (which can be either funded by insurance or out-of-pocket expenditure). Over the last decade, the use of the private sector facilities within NHS funded care has increased; the 2012 Health and Social Care Act allowed NHS hospitals to increase their private revenue and distribute contracts to private for-profit providers. Private providers are now thought to account for around 25 % of NHS spending [4,5]. More recently, the government has announced plans to expand commissioning to privately run diagnostic centres to improve waiting times with the addition of 13 extra community diagnostic centres to the 114 currently operating by 2025 [6].

Increasing strain on the public health care system has, and will continue to have, detrimental impacts on England's health inequalities which have been further entrenched by the pandemic [7]. Increasing the use of private healthcare separate from the NHS may result in the formation of two tiers of waiting times, whereby those who can afford out-of-pocket expenses or insurance, or who are employed by companies that provide health insurance, will benefit from quicker waiting times. In turn, this may impact other inequalities, such as an individual's ability to return to work.

Despite the increasing use of private healthcare, there are limited analyses of private healthcare patterns in England. A 2023 analysis of elective hospital care across the pandemic has highlighted that NHS-funded care provided by the private sector increased by almost two thirds (63 %) between the first and second year of the pandemic, compared to under a third (31 %) increase for care provided by the NHS, suggesting a slower recovery of NHS services post-pandemic [8]. Geographic differences in private care provision were also shown, with London and the southeast having the lowest proportion of NHS-funded care and highest proportion of privately-funded care in 2021/22 [8]. Assessing how these geographical patterns are changing over time is necessary to further understand and anticipate trends. Other governments around the world where public and private hospitals also work in parallel are facing similar challenges, for instance in Italy and Spain [9]. Spain has recorded increasing growth in the private health insurance industry between 2020 and 2023, with furter increases projected [10]. In Italy, and public hospitals face similar challenges to the NHS, such as staff recruitment, underinvestment, and understaffing [11,12].

We aimed to first describe the number and emerging post-pandemic trends in orthopaedic procedures by the NHS and private providers and NHS waiting lists from 2019 to 2023, and second examine the differences between the north and south of England. We focus on orthopaedic procedures as this is the speciality with the largest number of consultants working privately [13].

## 2 Methods

### 2.1 Study design and data sources

We undertook a retrospective observational analysis of national waiting times and private orthopaedic surgical procedure volumes between April 2019 and 2023 (between March 31st, 2019 and April 1st, 2023) and across England geographically.

This analysis was performed at Integrated Care Boards (ICBs) level, which represent 42 different geographical areas within England. For each ICB, the following quarterly information was obtained: the number of NHS-commissioned and privately funded privately delivered procedures, the population size, NHS orthopaedic waiting list time and which larger geographical areas the ICB was within.

Quarterly private surgical procedure volumes were obtained from the Public Health Information Network (PHIN). PHIN is a national publicly available dataset of all private procedure volumes by site and procedure in England. The dataset reports the total number of procedures in the preceding 12 months on a quarterly basis. If less than 12 months of data were recorded at a given site, results were adjusted to calculate predicted 12-month averages based on the data available (see Appendix Fig. 1 for the number of sites adjusted at each time point). Sites were grouped into their geographical ICB area. The total quarterly number of privately and NHS-funded privately delivered procedures in each ICB in the preceding 12 months was calculated. To account for population size, the number of procedures per head of population was calculated. A full list of included procedures can be found in Appendix Table 1.

The monthly incomplete pathways referral-to-treatment (RTT) Waiting Times Data 2022-23 published monthly from NHS England were obtained for each ICB. This measures the average weekly waiting time of the patients who are yet to start treatment at the end of each month. To match the procedure data, for each quarter, the mean value of the preceding 12 months was calculated.

To assess geographical differences, ICBs were grouped in two ways. First by regional areas (East, London, Midlands, South East, South West, North East, North West) before further analysis grouped these into the North (Midlands, North East, North West) and the South of England (East, London, South East, South West). The total number of individuals in a given area were obtained from NHS fingertips.

### 2.2 Data analysis

We performed descriptive analysis on surgical volumes and waiting times stratified by geographical location and time period. Independent variables were: the waiting list length, geographical area (either region or north-south), and time. All analysis was carried out using R.

## 3 Results

### 3.1 Procedure volumes

Between April 2019 and April 2023, there were a total of 1,469,450 procedures across the private sector. 1,030,095 NHS-funded privately delivered orthopaedic procedures and 439,355 privately funded orthopaedic procedures undertaken. This is equivalent to 42/10,000 people and 18/10,000 people per year respectively.

The most common orthopaedic privately delivered procedures performed were similar: the top three NHS-funded privately delivered procedures were knee replacement, hip replacement, and knee arthroscopy (Table 1) and the commonest privately funded were hip replacement, knee arthroscopy, and knee replacement.

**Table 1:** Top 10 most common orthopaedic procedures performed between April 2019–2023.

	NHS-funded privately delivered procedures		Privately funded privately delivered procedures^a^	
	Procedure Type	Number	Procedure Type	Number
1	Knee replacement	167,550	Hip replacement	82,080
2	Hip replacement	152,415	Knee arthroscopy	74,686
3	Knee arthroscopy	91,915	Knee replacement	63,254
4	Carpal tunnel release	74,740	Spinal decompression	28,696
5	Patella resurfacing	61,725	Subacromial joint decompression	22,108
6	Removal of metalwork from bone	53,450	Patella resurfacing	21,625
7	Spinal decompression	48,835	Carpal tunnel release	18,446
8	Subacromial joint decompression	35,730	Knee ligament reconstruction	17,931
9	Therapeutic spinal tap	34,390	Removal of metalwork from bone	11,519
10	Spinal facet joint denervation	25,790	Shoulder rotator cuff repair	11,079

a Includes out-of-pocket and insurance-based.

The south of England performed more privately delivered procedures per year compared to the north (63 vs 57/10,000 people) (Table 2); whilst there was almost double the number of privately funded privately delivered procedures (23 vs 12/10,000), there were fewer NHS-funded privately delivered procedures (40 vs 45/10,000 respectively).

**Table 2:** Mean number of privately delivered procedures per year between 2019 and 2023 in England.

Location	Number of private procedures/10,000 people		
	Total	NHS-funded privately delivered procedures	Privately funded privately delivered procedures^a^
North	57	45	12
Midlands	53	40	13
Northwest	58	47	11
Northeast	60	49	11
South	63	40	23
London	48	26	22
East	60	39	21
Southeast	72	47	26
Southwest	78	56	22

a Includes insurance and out-of-pocket funded.

The southwest undertook the most procedures per 10,000 compared to other regions. There was a large variation in the number of NHS-funded privately delivered procedures per 10,000 between regions; notably, London had less than half the number of procedures when compared to the Southwest (26 vs 56/10,000) despite having the same number of privately funded privately delivered procedures (22/10,000).

### 3.2 Waiting times

The north of England has consistently shorter waiting times than the south; patients waited approximately 0.9 weeks less pre-pandemic, and 0.8 weeks less post-pandemic (Fig. 1). Waiting times in both areas more than doubled from April 2020 to April 2021. After a slight decrease for approximately 6 months, the waiting times plateaued before a slight divergence between the two areas starting at the end of 2022 when waiting times in the north declined.

**Fig. 1:**
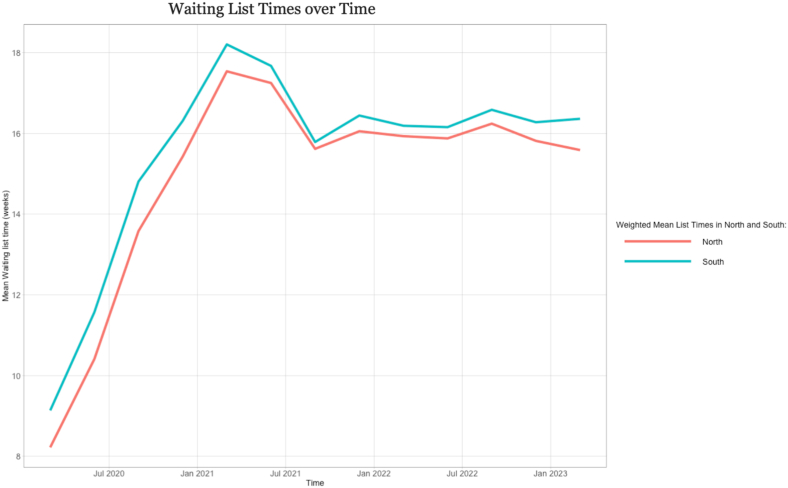
Time trends in waiting list times (months) for North and South of England.

There was marked variation between regions in waiting time over time (Table 3); there was a five-week difference in waiting time between the longest (the east at 18.7 weeks) and the shortest waiting time (the northeast at 13.7 weeks). The east had the longest waiting times before and after the pandemic at 9.9 weeks and 18.7 weeks respectively. There were also varied percentage changes before and after the time period, ranging from 55 % (London) to 105 % (midlands).

**Table 3:** NHS mean waiting times (weeks) between 2019 and 2023 in England.

Location	April 2019–2020	April 2022–2023	Change over time
North	8.2	15.6	+90 %
Midlands	8.0	16.5	+105 %
Northwest	7.7	15.4	+99 %
Northeast	8.4	13.7	+63 %
South	9.1	16.4	+79 %
London	8.9	13.9	+55 %
East	9.9	18.7	+88 %
Southeast	8.5	14.9	+75 %
Southwest	9.4	17.1	+82 %

### 3.3 Time trend of NHS waiting times and privately and NHS-funded privately delivered procedures

**Fig. 2a and b:**
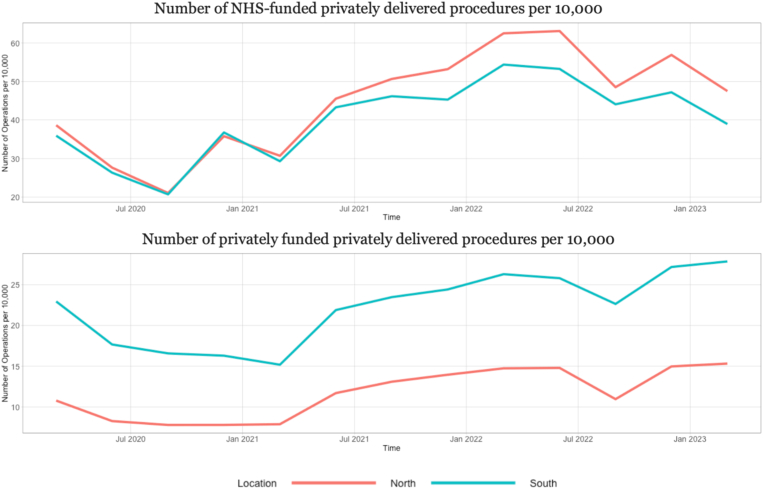
Number of privately and NHS-funded privately delivered procedures per 10,000 people by North-South.

Regarding the number of NHS-funded privately delivered procedures, it appears that the difference in numbers between the North and South increased over the period 2021–2023 (Fig. 2a). The south has had a consistently higher number of privately funded privately delivered procedures when compared to the North (Fig. 2b).

**Fig. 3a and b:**
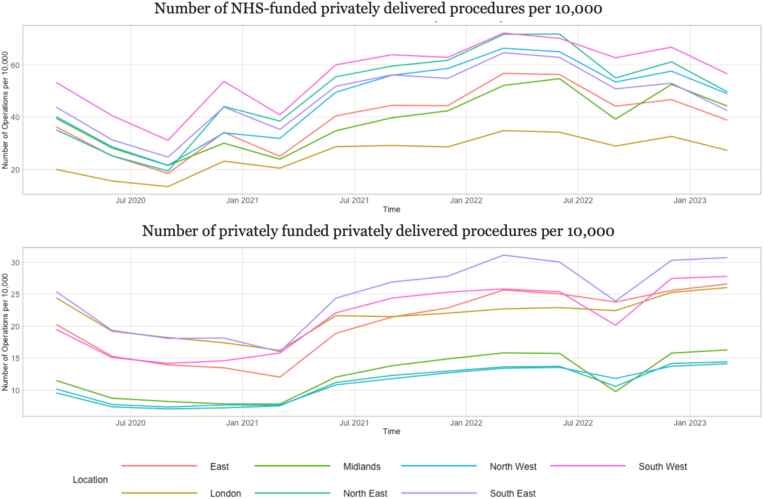
Number of privately and NHS-funded privately delivered procedures per 10,000 people by region.

Across the time period, all four Southern regions had higher numbers of privately funded privately delivered procedures per 10,000 of the population when compared to Northern regions (Fig. 3b). Regarding NHS-funded privately delivered procedures, London appeared to have a considerably lower number per 10,000 compared to other regions.

## 4 Discussion

### 4.1 Summary

Following the pandemic, waiting times and numbers of privately delivered procedures have increased. The trends in private healthcare funding and waiting times have considerable geographic inequalities; the north of England has shorter NHS waiting times, possibly linked to the fact it has a higher number of NHS-funded privately delivered procedures but also due to wider population or organisational factors. The use of NHS-funded privately delivered procedures may also contribute, although it is not possible to quantify this using data presented in this article. These changes may also reflect the increases in underlying demand from both the NHS-funded and self-paying population. The south of England has double the number of privately funded privately delivered procedures per 10,000 compared to the north, yet still face longer waiting times. This higher number of privately funded privately delivered procedures could be due to higher wealth, more opportunities, different demographics and wider organisational factors. These healthcare patterns indicate that there is already an emergence of a two-tier system, particularly in the south, whereby more people than the north are privately funding procedures, and those who cannot afford to pay privately are more likely to face long NHS waiting times than in the north. Importantly, it should be acknowledged that Covid-19 caused significant delays to elective procedures, and these results could be reflective of ongoing regional variation in backlof demand from this period.

### 4.2 A two-tier system

The increase in waiting times and geographic variation in private health care use raises concerns. One study found that while regional differences in the provision of hip and knee replacement across the NHS and private sector have reduced over time, socioeconomic inequalities remain, particularly in the private sector [14]. While private healthcare might not keep increasing due to capacity reasons, it is possible that an increase in demand could lead to further private sector expansion. Increased private health care use may reduce demand for NHS services, however there may be unintended consequences, such as a reduction in surgeons undertaking NHS procedures.

Orthopaedic procedures have a myriad of health benefits including reduced pain, improved mobility, and subsequent knock-on effects for wider, already unequally distributed health outcomes, such as opioid usage, independence, employment, and mortality. Furthermore, delays in procedures can worsen outcomes; knee replacement procedures delayed by over 6 months is projected to increase the odds of poor outcome by 50 % [15]. Inequalities already exist in orthopaedic procedures, with more hip and knee replacements being carried out in the least disadvantaged groups of people, with the difference seen most in older populations [14]. While socioeconomic inequality exists in the NHS and private sector for hip replacement, a significant difference is only seen for knee replacement in the private sector [14].

### 4.3 North-south divide

Longer NHS times are disproportionately affecting the south, despite a consistently higher number of privately funded privately delivered procedures per 10,000 people. This could be due to out-of-pocket expenditure as a result of patients not wanting to wait for the longer waiting time and/or due to higher numbers of people with insurance in the south. In the Australian setting, private insurance coverage is used in parallel to public services to reduce waiting times, although only with a small effect size, suggesting the increasing coverage seen in the UK is unlikely to alleviate times further [16]. Subsequently, without tackling health care capacity problems or increasing the number of NHS-funded privately delivered procedures these waiting times might not decrease. It is worth mentioning that supply side factors may be contributing to the lower numbers of NHS funded privately delivered procedures in southern regions, for instance in London where numbers are the lowest. Private providers in regions where populations are able to privately fund procedures, or have healthcare insurance, may be less willing to take NHS-funded patients. The discrepancy between NHS-funded privately delivered procedures and waiting times has the potential to exacerbate health inequities, particularly within the south.

These findings contrast the overall patterns in health outcomes between the north and south, whereby the north is known to experience negative effects of inequalities with higher rates of morbidity (including reporting good health, highest percentage of people reporting disability limiting daily life) and mortality [17].

### 4.4 Regional differences

The unequal lengths of waiting times and funding are more complex than simply a north-south divide. There are differences in the number of NHS-funded privately delivered procedures regionally, particularly in terms of London. London has the second shortest waiting time and a significantly larger number of privately funded privately delivered procedures. The number of NHS-funded privately delivered procedures is less than half that of the southwest. Capacity of and demand for private healthcare is higher in London and the southeast with 48 of 190 private hospitals located in greater London [18].

### 4.5 Strengths and limitations

This analysis provides a novel insight into the time trends of private healthcare use across England. We were only able to examine data at the local health system (ICB in England) level because data is not comparable at a hospital or trust level. The average local health system is 1.5 million people, but ranges from about 500,000 to 3 million. Therefore there could be substantial inequalities within an individual health care system which we did not identify. However, patients are usually willing to travel for secondary and tertiary care, so we believe the comparison is useful. We only assessed orthopeadic procedures because of difficulties in comparing cross-speciality NHS waiting time data with private procedure data. However, these are the largest proportion of procedures and reflect wider attitudes to willingness to pay for private health care.

### 4.6 Implications for research, practice, and policy

Private outsourcing has been trialled before within health care in the UK with varied success. In the 2000s, the government used the private sector as a tool to reduce waiting lists, driving the proportion of private healthcare funding to be higher than it currently is now [19]. This was associated with a progressive distribution of funds, which resulted in a relative increase of funding becoming available for areas with higher levels of deprivation [19], in addition to a range of ambitious policies which aimed to reduce health inequalities.

After the 2012 Health and Social Care Act in the UK, an analysis of private healthcare found that for each percentage point of outsourcing to the private sector, there was a corresponding annual increase in treatable mortality by 0.38 % (95 % CI 0.22–0.55, p = 0.0016) [20]. A similar pattern was seen in Italy [21]. Worsening outcomes associated with higher use of outsourcing to the private sector could be due to deteriorating quality of care as the focus is shifted to cost-cutting and profit over patient care This shift can potentially impact staffing and thus quality of care. Over the last three years, staff satisfaction with staffing levels and pay has decreased in the NHS, and increasing numbers are striking and considering leaving [22]. Staff shortages and dissatisfaction may lead to staff moving towards private health care organisations.

### 4.7 Conclusion

We found considerable geographical inequalities of access to orthopaedic private healthcare between regions within England, with a potential emergence of a two-tier healthcare system. Simply relying on the private sector to reduce waiting lists, without oversight, may exacerbate regional and socioeconomic differences. Policymakers should consider how the unequal distribution of funding and NHS-funded privately delivered orthopaedic procedures could perpetuate inequalities.

## Funding

This research did not receive any specific grant from funding agencies in the public, commercial, or not-for-profit sectors.

## Declaration of competing interest

The authors declare that they have no known competing financial interests or personal relationships that could have appeared to influence the work reported in this paper.

The author is an Editorial Board Member/Editor-in-Chief/Associate Editor/Guest Editor for *Public Health in Practice* and was not involved in the editorial review or the decision to publish this article.

## Figures and Tables

**Fig. 1:**
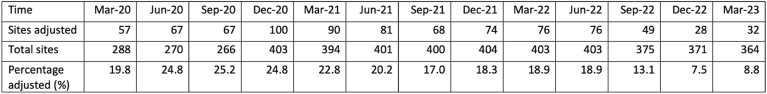
Number of sites adjusted for each timepoint.

**Table 1:** List of included procedures

Procedure Group ID	Procedure Group
3	Carpal tunnel release
4	Cubital tunnel release
84	Subacromial joint decompression
116	Dupuytren's contracture surgery
119	Dupuytren's contracture surgery (revision)
121	Bursa excision (surgical)
122	Bursa treatment (non-surgical)
123	Trigger finger release
124	Shoulder rotator cuff repair
127	Spinal facet joint denervation
129	Forefoot reconstruction (complex)
130	Hindfoot reconstruction
131	Forefoot reconstruction (simple)
132	Removal of metalwork from bone
133	Hip replacement (primary)
134	Hip replacement (revision)
135	Knee replacement (primary)
136	Knee replacement (revision)
138	Great toe fusion
140	Knee ligament reconstruction
141	Frozen shoulder release
142	Tennis elbow release
143	Bunion correction
144	Knee arthroscopy
147	Manipulation of joint
148	Shoulder replacement (primary)
156	Hip arthroscopy
157	Shoulder arthroscopy
158	Ankle arthroscopy
159	Elbow arthroscopy
160	Wrist arthroscopy
161	Foot arthroscopy
168	Ankle ligament reconstruction
169	Ankle replacement (primary)
170	Ankle replacement (revision)
175	Debridement of joint (other)
176	Elbow replacement (primary)
177	Elbow replacement (revision)
182	Knee replacement (primary - unicompartmental)
184	Metatarsal osteotomy
191	Spinal nerve root procedure
207	Shoulder replacement (revision)
210	Spinal decompression (lumbar)
212	Spinal decompression (revision lumbar)
213	Spinal decompression (cervical)
214	Spinal decompression (revision cervical)
216	Spine manipulation
220	Removal of spinal instrumentation or fixation
221	Spinal fusion (lumbar/thoracic)
222	Spinal fusion (cervical)
223	Spinal fusion (revision)
224	Spinal disc replacement
225	Spinal deformity correction (scoliosis)
226	Spinal spacer insertion
227	Knee osteotomy
235	Shoulder stabilisation
237	Acromioclavicular joint stabilisation
239	Sternoclavicular joint decompression
240	Shoulder slap repair
251	Shoulder replacement (primary - reverse polarity)
252	Shoulder replacement (revision - reverse polarity)
260	Elbow stabilisation
262	Elbow decompression
267	Other toe fusion
268	Spinal fusion (anterior cervical)
269	Spinal decompression (posterior cervical)
270	Cervical corpectomy
271	Cranio-cervical decompression
272	Spinal fusion (revision anterior cervical)
273	Spinal decompression (revision posterior cervical)
274	Spinal decompression (thoracic)
275	Spinal decompression (revision thoracic)
276	Spinal fusion (revision lumbar)
277	Spinal decompression (lumbar/thoracic)
278	Spinal decompression (revision lumbar/thoracic)
279	Spinal fusion (lumbar)
280	Spinal disc replacement (cervical)
281	Spinal disc replacement (lumbar)
282	Spinal fusion (cervical C1/C2)
283	Spinal fusion (posterior cervical)
284	Spinal fusion (cranio-cervical)
429	Spinal fracture decompression
430	Spinal lesion excision
431	Spinal operation (other)
432	Spinal stabilisation
447	Repair of fractured neck of femur
448	Hip dislocation reduction
449	Hip joint manipulation
450	Head of femur replacement
451	Head of femur replacement (revision)
452	Excision foot bone
457	Correction of congenital foot abnormality
458	Shoulder reconstruction
459	Sternoclavicular joint arthroscopy
460	Acromioclavicular joint arthroscopy
461	Acromioclavicular joint decompression
462	Knee replacement (primary - patellofemoral)
464	Tendon sheath release (wrist)
465	Release nerve at wrist
468	Hand reconstruction
469	Removal carpal bones
470	Hand amputation
472	Congenital deformity of hand repair
597	Spinal cord lesion removal
598	Spinal cord neurostimulator procedure
600	Spinal cord procedure
601	Spinal fluid drainage
602	Therapeutic spinal tap
655	Facial nerve disorder surgery
664	Lower limb amputation
688	Ankle Fusion
694	Pelvic Osteotomy
696	Patella resurfacing
